# Vanishing bile duct syndrome with hyperlipidemia after ibuprofen therapy in an adult patient: a case report

**DOI:** 10.1186/s12876-018-0869-9

**Published:** 2018-09-29

**Authors:** Wen Xie, Qi Wang, Yuanjiao Gao, Calvin Q. Pan

**Affiliations:** 1 Center of Liver Diseases, Beijing Ditan Hospital, Capital Medical University, Beijing, China; 2 Division of Gastroenterology and Hepatology, Department of Medicine, NYU Langone Health, New York University School of Medicine, 132-21 41Ave, Flushing, New York 11355 USA

**Keywords:** Jaundice, Ibuprofen, Drug-induced liver injury, Vanishing bile duct syndrome, Hyperlipidemia

## Abstract

**Background:**

Non-steroidal anti-inflammatory drugs (NSAIDs) are frequently prescribed drugs and can cause drug-induced liver injury. Although patients with drug-induced liver injury from NSAIDs often recover spontaneously, 3% of them required hospitalization and those with persistent cholestasis present a diagnostic challenge. Recently, a few cases of children with persistent jaundice reported have been linked to the vanishing bile duct syndrome. However, data on adult patients is limited.

**Case presentation:**

We report herein a case of an adult patient who had persistent cholestasis with hyperlipidemia from the VBDS after ibuprofen use. We described a female patient with severe jaundice after taking ibuprofen, although she had no history of liver disease before. The drug-induced liver injury from ibuprofen was identified by clinical features and liver biopsy, which included the Roussel Uclaf Causality Assessment Method scores of 6 and pathological features of cholestasis with stage four drug-induced injury as well as loss of bile duct structures. The clinical course was featuring with persistently high levels of bilirubin associated with hyperlipidemia over the period of one month, although the laboratory abnormalities were slightly improved spontaneously after the cessation of ibuprofen. Her autoantibodies markers including AMA-M2 ASMA, RO-52, LKM, SLA, and anti-glycoprotein-210 were negative. The second liver biopsy was performed on day 213 due to persistent hyperbilirubinemia. Pathological findings were consistent with the diagnosis of vanishing bile duct syndrome.

**Conclusions:**

A rare case of ibuprofen-associated vanishing bile duct syndrome in an adult female patient is presented. Clinicians need to be aware of vanishing bile duct syndrome as a serious consequence of ibuprofen use in adult patients, although ibuprofen is considered to be among the safest NSAIDs.

## Background

Drug-induced liver injury (DILI) is a term used to describe a spectrum of clinical presentations and severity that ranges from a mild elevation of liver enzymes to acute liver failure and death. The severe form of DILI leads to a long-term liver-related morbidity and mortality in 1% to 3% of cases [1]. Non-steroidal anti-inflammatory drugs (NSAIDs) are frequently prescribed drugs and can cause DILI [2]. Although patients with DILI from NSAIDs often recover spontaneously, 3% of them required hospitalization and those with persistent cholestasis present a diagnostic challenge. Ibuprofen is a widely used antipyretic and analgesic NSAID. However, cases with sub-fulminant hepatitis requiring liver transplantation following ibuprofen overdose have been reported [3]. It has been suggested that ibuprofen can produce an unpredictable, idiosyncratic, type B reactions as a hypersensitivity reaction (HSR) in susceptible individuals [4]. The true HSR is a systemic disease defined by the triad of fever, rash, and internal organ involvement that starts 1 day to 12 weeks after the initiation of therapy [4]. Recently, several cases of children with persistent jaundice after taking ibuprofen have been linked to liver injury as the targeting organ damage from HSR and developed the vanishing bile duct syndrome (VBDS) [5–8]. However, data on adult patients is limited.

## Case presentation

A woman in her 40s presented with acute onset of marked jaundice that had become progressively worsening over the course of 30 days, after taking ibuprofen intermittently for menalgia. The associated symptoms included profound fatigue and dark urine. No other symptoms were present. Twelve months prior to the onset of jaundice, she had menorrhagia after receiving the diagnosis of adenomyosis of uterus. She started only on ibuprofen 300 mg bid by mouth for 2–3 days each month with a total of six months when menalgia occurred. Her medical history included type II diabetic for one year on oral acarbose 50 mg TID and metformin 500 mg three times daily. She had no other medications. She had a surgical resection for a right ovarian cyst about 20 years ago. At the time, she was a non-smoker and did not consume any alcoholic drinks or recreational drugs. Clinical examination revealed normal vital signs and mental status. Although she has scleral icterus and a soft, non-tender abdomen with a surgical scare, neither signs of ascites nor hepatomegaly were presented. Her spleen was palpable at 3 cm below the left costal margin. There was no asterixis.

### Investigations

**Fig. 1:**
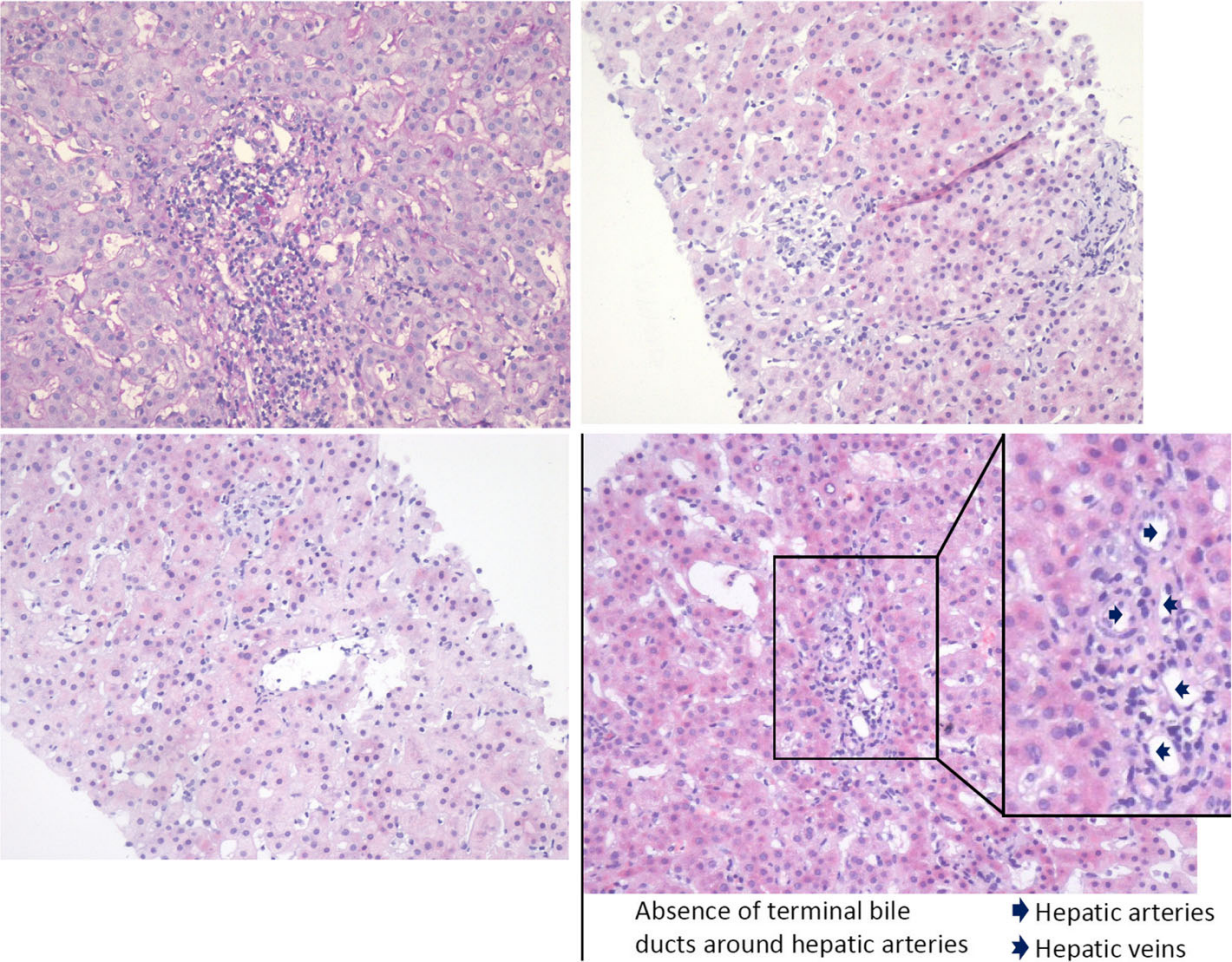
The First Liver-Biopsy Specimens on Day 28. The specimen revealed seven peri-portal areas and lobular plates was intact. Hepatic lobule scattered moderate necrosis with granuloma formation and inflammatory infiltration around central veins. There were mild sinusoidal expansion with a small amount of sinus lymphocytes and eosinophil infiltration although a small quantity of DPAS-positive macrophages was present. In peri-portal area, mild to moderate expansion with mixed inflammatory cell infiltration including lymphocytes and eosinophils were noted. Eosinophilic changes were present in hepatocyte cytoplasm. In portal areas, there were accumulation of epithelium-like cells and interstitial cells, fibrous tissue hyperplasia, and interstitial fibrosis. In addition, interlobular bile duct injury with loss of bile duct structure around hepatic arteries was noticed in more than 50% of lobules. Bile salt deposition was visible among peripheral hepatocytes. The results of Immunohistochemistry stain were the followings: CD10 (+), CD38 (+), CK19 (+), CK7 (+), HBcAg (−), HBsAg (−), HCV (−), Mum-1 (+), Pre-S1 (−), ubiquitin (−); Patent staining results: DPAS (−), Masson (+), PAS (−), reticulocyte staining (+), copper and iron staining (−), rhodanine (−). Ishak grading:necroinflammatory activity score of 5 and fibrosis score of 2. The clinical implications of special markers are the followings: CD10 is a maker of bile canaliculus; CD38 and MUM1 are plasmacytic markers; CK7 and CK19 are biliary markers. CK7-positive hepatocytes indicate cholestatic hepatic changes

Laboratory testing revealed a normal completed blood count except Hb of 82 g/L (110–150); normal plasma thromboplastin antecedent and partial thromboplastin time; deranged liver function tests (alkaline phosphatase 1598 U/L, alanine transaminase 207 U/L, aspartate transaminase 247 U/L, total bilirubin 103 umol/L with direct bilirubin 75 umol/L, and albumin 30 g/L); abnormal lipid profile (total cholesterol 43 mmol/L, triglyceride 3.6 mmol/L, high-density lipoprotein cholesterol 6.4 mmol/L, low-density lipoprotein cholesterol 35 mmol/L Apolipoprotein-A1 0.6 g/L, Apolipoprotein-B 1.2 g/L); and normal electrolytes except potassium of 3.1 mmol/L. Viral serology was negative for hepatitis A, B, C, and Epstein-Barr virus. Antibody tests for hepatitis E, ASMA, RO-52, LKM, AMA, AMA-M2, SLA, and gp210 were negative. Her ceruloplasmin, ferritin, and iron were normal. The titers of cytoplasmic type and nuclear membrane type of ANA were 1:100 and 1:320, respectively. Her IgG level was 15.9 g/L and cytomegalovirus (CMV) PCR was negative although CMV IgG was > 500.00 U/ml. The patient was admitted and ibuprofen was discontinued. Further investigations included the followings: an MRCP revealed stones in the gallbladder without intrahepatic or extrahepatic bile duct dilatation; a computed tomography scan with contrast on day 10, which showed a few small enhanced patchy lesions on the left hepatic lobe likely due to the abnormal perfusion, mild splenomegaly, but no vascular abnormalities or intraperitoneal free fluid. However, three follow-up MRI exams with contrast on days 100, 185 and 260 showed a normal size of the spleen and normal diameters of both intra/extra-hepatic ducts. There were no signs of lymphoma. On the day of first evaluation, the Roussel Uclaf Causality Assessment Method score (RUCAM) was 6 (*R* = 0.32, grade III liver injury). A liver biopsy was performed on day 28 from the onset of her jaundice. The pathology slides were presented in Fig. 1, which revealed biliary injury and absence of small terminal bile ducts around hepatic arteries affecting over 50% of sampled portal tracts. In addition, Bile salt deposition was visible among peripheral hepatocytes with no evidence of steatohepatitis or significant fibrosis. The Ishak grading showed necroinflammatory activity score of 5 and fibrosis score of 2. The findings were consistent with DILI and VBDS.

### Differential diagnosis

This 40-year-old woman, who had a history of taking ibuprofen, became acutely ill with a rapid progressive jaundice and high cholesterol followed by profound fatigues that developed over a 4-week period. The differential diagnosis included drug-induced liver injury, viral hepatitis, marker-negative autoimmune hepatitis, non-alcoholic steatohepatitis, overlap syndrome, primary sclerosing cholangitis (PSC), and primary biliary cholangitis (PBC). Her initial presentations were consistent with intrahepatic cholestasis. She had laboratory evidence of acute hepatic injury and liver biopsy suggested drug-induced liver injury. Moreover, the histological features also suggested VBDS. Thus, further differential diagnosis for VBDS was needed, which includes not only aforementioned drug-induced liver injury, [9] viral hepatitis, autoimmune hepatitis; but also biliary obstruction, idiopathic adulthood ductopenia, Alagille syndrome, PSC, PBC, lymphoma, and ischemic liver injury [10].

### Clinical diagnosis

As supported by the clinical data and the RUCAM score of 6, which indicated modestly probability of DILI with severe liver injury (stage III), her clinical diagnosis was an ibuprofen-induced liver injury resulting on persistent cholestasis and hyperlipidemia. The pathology diagnosis was DILI at the stage of IV. In addition, the features of bile duct injury and the loss of bile duct structures were consistent with VBDS. She has no hepatic duct dilatation or signs of lymphoma in MRI study on day 260. In addition, her negative test results of AMA-M2 and other autoantibodies did not support the diagnosis of PBC or PSC.

### Clinical course, treatment, and outcomes

**Fig. 2:**
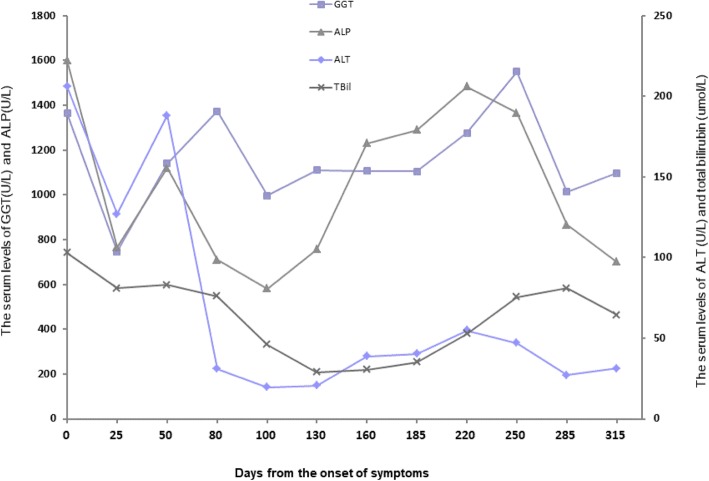
Changes of Biochemistry Parameters after the Onset of Symptoms. The hyperbilirubinemia persisted with normal PT during the period of patient’s outpatient visits, although the levels were slowly trending down from over 100 umol/L to 30 umol/L. Thereafter, the patients’ ALT levels were fluctuated at the range of 70–180 U/L. GGT = gamma-glutamyltransferase (U/L); ALP = alkaline phosphatase (U/L); ALT = alanine aminotransferase (U/L); and Tbil = total bilirubin (umol/L)

**Fig. 3:**
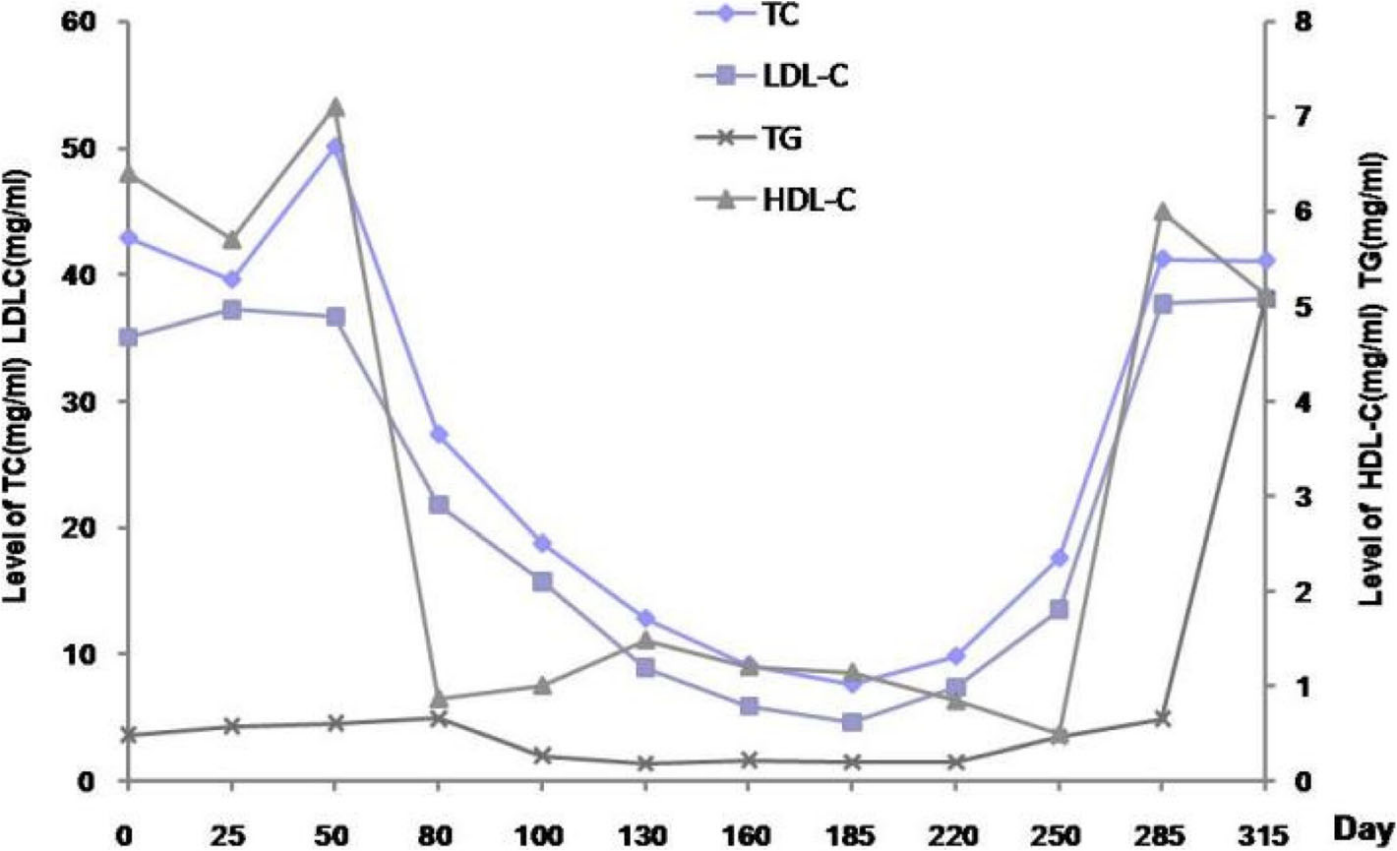
Changes of Lipid Profile after the Initial Presentation. The patient had persistent hyperlipidemia during the entire observational period. Her total cholesterol and triglyceride levels both remained at levels above 10 times higher than normal, whereas the low-density lipoprotein cholesterol and high-density lipoprotein cholesterol levels were always below 10 mmol/L. TC = total cholesterol (mg/ml); LDL-C = low-density lipoprotein cholesterol (mg/ml); TG = triglycerides (mg/ml); and HDL-C = high-density lipoprotein cholesterol (mg/ml)

**Fig. 4:**
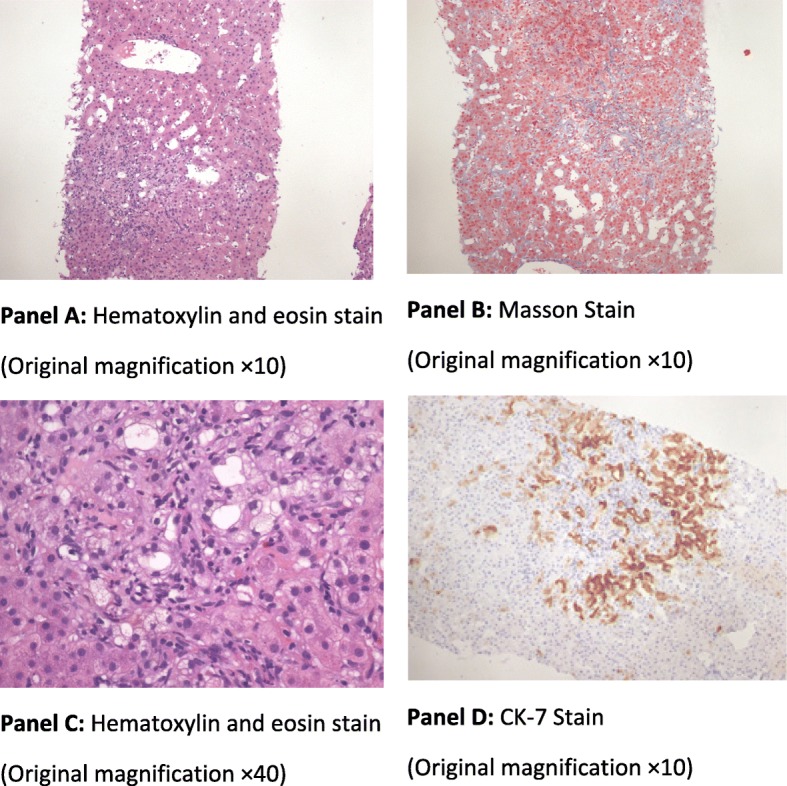
The Second Liver-Biopsy Specimens on Day 213. The second liver biopsy indicated again the absence of small terminal bile ducts. The specimen shows six peri-portal areas and lobular plates were intact. Hepatic lobule scattered with focal moderate necrosis and small granuloma formation. The cytoplasm of hepatocytes was loose with eosinophilic changes and inflammatory infiltration around central veins. There were mild sinusoidal expansion with a small amount of sinus lymphocytes and eosinophil infiltration. There were bile duct injuries, a loss of bile duct structure around central arteries with visible lymphocytes, and a small amount of plasma cell infiltration. In addition, there was mild interface inflammation, interstitial fibrous tissue hyperplasia, and peripheral hepatocyte bile salt deposition. Immuno-histochemical stains were positive for CD10, CD38, CK19, CK7, Mum-1, Masson, and Copper-rhodanine. However, the stains were negative for HBcAg, HBsAg, PRE-S1, ubiquitin, D-pas, PAS, and iron. Ishak grading:necroinflammatory activity score of 4 and fibrosis score of 2. There are four panels inside the image of Fig. 4 including panel A for the specimen with hematoxylin and eosin stain (manification x 10), panel B for the specimen with Masson stain (manification x 10), panel C for the specimen with hematoxylin and eosin stain (magnification x 40), and panel D for the specimen with CK-7 stain (magnification x10)

Due to further deterioration of liver function tests despite the cessation of ibuprofen, the patient was hospitalized and received supportive care with intravenous therapy of polyene phosphatidylcholine 930 mg daily. Her oral medication included silibinin capsule at the dose of 200 mg daily, glutathione 2.4 g daily, and weight-based ursodeoxycholic acid at the dose of 250 mg three times a day. In addition, she continued on acarbose and metformin for her diabetic. Although she had progressively worsening jaundice over the first 7 days, the patient’s fatigue and biochemistry were subsequently improved with her total bilirubin decreased from167 umol/L to 130 umol/L. She had normal prothrombin time (PT) during her hospital stay. She was discharged on day 47 and followed up in outpatient’s clinic with the last visit on day 315. Her hyperbilirubinemia persisted with normal PT during the period of her outpatient visits, although the levels were slowly trending down from over 100 umol/L to 30 umol/L. The changes in biochemistry parameters including bilirubin and alanine aminotransferase (ALT) are shown in Fig. 2. Her ALT levels fluctuated at the range of 70–180 U/L. In addition, the patient had persistent hyperlipidemia during the entire observational period. Her total cholesterol and triglyceride levels both remained at levels above 10 times higher than normal, whereas the low-density lipoprotein cholesterol and high-density lipoprotein cholesterol levels were always below 10 mmol/L (Fig. 3). Owing to the persistent elevation on the serum levels of bilirubin and ALT, a second liver biopsy was performed on day 213. Pathology report indicated again the absence of small terminal bile ducts, interstitial fibrous tissue hyperplasia, bile salt deposition in the peripheral liver cells, and visible lymphocytes with small amount of plasma cell infiltration; Ishak necroinflammatory activity score of 4 and fibrosis score of 2; keeping with a diagnosis of acute VBDS but some features of autoimmune hepatitis (Fig. 4).

## Discussions and conclusions

VBDS is a severe cholestatic disease associated with toxic effects of medications [9, 11]. As a complication of acute drug-induced liver injury, VBDS generally manifests 1 to 6 months after the onset of the liver injury [9]. Several diseases or syndromes as the causes for VBDS have been discussed in the section of differential diagnosis. Although DILI is an uncommon etiology for the VBDS, several medications have been reported to be related to the development of VBDS after liver injury. These medications include: antifungals or antibiotics like terbinafine, [12] meropenem, [13] and azithromycin; [11] anti-seizure medications such as valproic acid, [14] carbamazepine, [15] and lamotrigine; [16] and NSAIDs such as loxoprofen, diclofenac, and ibuprofen in pediatric cases [5–8, 17]. Five cases of ibuprofen-induced liver injury with the complication of VBDS have been reported by far and all were children. The first case was reported by Alam at el in 1996, which indicated that VBDS was temporally associated with ibuprofen [8]. Subsequently, Kim et al. reviewed three cases of VBDS from ibuprofen-induced liver injury in patients with ages ranging from 9 months to 10 years old [7]. Among them, two had completed clinical and biochemical recovery in 4 to 7 months after the onset of VBDS; one had persistent jaundice and required transplant evaluation. In a case recently reported by Bastuck et al. VBDS occurred in a 7-year-old child who had toxic epidermal necrolysis after oral ibuprofen intake. However, the patient had a complete recovery within 8 months [6]. These reports suggested that ibuprofen can cause acute VBDS, and weight-based ursodeoxycholic acid was commonly used for VBDS with supportive care, although steroids, immunosuppressive agents, or plasmapheresis were provided occasionally [12, 18]. Similar to the clinical presentations described in children, our case had acute onset of jaundice and VBDS developed approximately at weeks 4–6 from the ibuprofen-induced liver injury. However, unlike the outcomes of completed recovery in the majority of cases reported before, our patient had no significant improvement in biochemistry after a 10-month follow-up. Such different outcomes may be related to the lower liver stem cell or progenitor cell activity in the adult or aging liver when comparing to those in children [19]. In addition, hyperlipidemia was presented in our case. Although hyperlipidemia is an uncommon presentation in VBDS patients, it has been reported in a few pediatric patients. In the case presented by Basturk et al. the child was treated with supportive care, an steroid, and ursodeoxycholic acid, with complete normalization of lipid profile in 8 months [6]. Another case reported by Cho et al. was a 7-year-old boy with VBDS from trimethoprim-sulfamethoxazole combination therapy induced liver injury [20]. At the onset of VBDS, the patient’s total cholesterol level was 490 mg/dL and was improved to 385 mg/dL after nine weeks of ursodeoxycholic acid therapy (30 mg/kg/day) and returned to the normal range after one year. Lastly, Kim et al. reported a 7-month-old infant with ibuprofen associated toxic epidermal necrolysis, followed by severe and rapidly progressive VBDS [7]. She had a total cholesterol level of 760 mg/dL but recovered totally with supportive care in three months. The mechanism of hyperlipidemia in VBDS has not been fully understood. It has been suggested that cholestasis might affect cholesterol metabolism and lead to the formation of lipoprotein X, which is frequently mistaken for LDL on routine clinical tests [21]. Further studies are needed to explore the implications of hyperlipidemia in the setting of VBDS.

In summary, we report herein a case of an adult patient who had persistent cholestasis from the vanishing bile duct syndrome after ibuprofen use. The highlights of clinical features include acute onset of jaundice and severe hepatic impairment required hospitalization, followed by a very slow recovery with persistence of hyperbilirubinemia and hyperlipidemia. The clinical course differed from those previously reported in children, which was a completed clinical and biochemistry recovery. Clinicians need to be aware of VBDS as a serious consequence of ibuprofen use in adult patients, although ibuprofen is considered to be among the safest NSAIDs.

## Abbreviations

ALP: Alkaline phosphatase
ALT: Alanine aminotransferase
AST: Aspartate transaminase
CMV: Cytomegalovirus
DILI: Drug-induced liver injury
GGT: Gamma-glutamyltransferase
HDL-C: High-density lipoprotein cholesterol
HSR: Hypersensitivity reaction
LDL-C: Low-density lipoprotein cholesterol
NSAIDs: Non-steroidal anti-inflammatory drugs
RUCAM: Roussel Uclaf Causality Assessment Method
Tbil: Total bilirubin
TC: Total cholesterol
TG: Triglycerides
VBDS: Vanishing bile duct syndrome
